# Detecting sulphate aerosol geoengineering with different methods

**DOI:** 10.1038/srep39169

**Published:** 2016-12-15

**Authors:** Y. T. Eunice Lo, Andrew J. Charlton-Perez, Fraser C. Lott, Eleanor J. Highwood

**Affiliations:** 1 Department of Meteorology, University of Reading, Reading RG6 6BB, UK; 2 Met Office Hadley Centre, FitzRoy Road, Exeter EX1 3PB, UK

## Abstract

Sulphate aerosol injection has been widely discussed as a possible way to engineer future climate. Monitoring it would require detecting its effects amidst internal variability and in the presence of other external forcings. We investigate how the use of different detection methods and filtering techniques affects the detectability of sulphate aerosol geoengineering in annual-mean global-mean near-surface air temperature. This is done by assuming a future scenario that injects 5 Tg yr^−1^ of sulphur dioxide into the stratosphere and cross-comparing simulations from 5 climate models. 64% of the studied comparisons would require 25 years or more for detection when no filter and the multi-variate method that has been extensively used for attributing climate change are used, while 66% of the same comparisons would require fewer than 10 years for detection using a trend-based filter. This highlights the high sensitivity of sulphate aerosol geoengineering detectability to the choice of filter. With the same trend-based filter but a non-stationary method, 80% of the comparisons would require fewer than 10 years for detection. This does not imply sulphate aerosol geoengineering should be deployed, but suggests that both detection methods could be used for monitoring geoengineering in global, annual mean temperature should it be needed.

Stratospheric aerosol injection (SAI) is one of the proposed solar radiation management (SRM) methods for limiting global surface temperature rise^1^,^2^. It involves deliberate injections of sulphate aerosols into the stratosphere to reduce incoming solar radiation. The natural analogue of SAI is large volcanic eruptions. In 1991, Mount Pinatubo erupted 20 Tg of sulphur dioxide (SO_2_) into the stratosphere, resulting in ~0.5 °C global cooling in 1992^3^. While potential side effects exist^4^,^5^,^6^, SAI scores high for effectiveness, affordability and timeliness in a preliminary analysis of the Royal Society^7^. Despite ethical concerns and doubts about the comprehensiveness of Royal Society’s tentative assessment^8^, some view SAI as a temporary ‘quick fix’ to undesirable climate warming in case aggressive conventional mitigation targets are not met.

In the event of SAI deployment, there would need to be a way to confirm it was actually reducing surface temperature and to look for its consequences. A drop in surface temperature after deployment does not necessarily mean SAI is working, as climate variability plays an important role in temperature fluctuations. This means we must be able to separate the forced changes due to SAI from climate variability, akin to the familiar ‘detection’ of historical anthropogenic climate change. A step further would be to attribute the changes observable with geoengineering to SAI in relation to other forcings. This would be necessary if we wanted to limit global warming to a more desirable rate or surface temperature to be under a particular value above pre-industrial levels via explicit feedback and management^9^,^10^. In other words, we would need to know how much of the observed temperature change is due to SAI in order to work out the amount or location of aerosol injection for the year following, so that our climate objectives for the future could be met.

Attribution can only be achieved with detection. This study aims to investigate how sensitive our ability to detect the influence of SAI on future annual-mean global-mean near-surface air temperature (SAT) time series is to two different detection methods and three data filtering techniques. Global-mean temperature is chosen because global cooling is likely to be the primary aim of SAI. It is a simple climate variable that the media and public are most interested in concerning geoengineering, and it has a high signal-to-noise ratio for detection due to averaging. Other climate variables may be used to detect a geoengineering response, but they are out of the scope of this study.

We assume a future geoengineering scenario, G4 from the Geoengineering Model Intercomparison Project (GeoMIP)^11^, which involves daily injections of SO_2_ into the stratosphere (16–25 km) at a rate equivalent to 5 Tg yr^−1^ during the period 2020 to 2070, in addition to the Representative Concentration Pathway of greenhouse gases that leads to 4.5 Wm^−2^ increase in radiative forcing in year 2100 relative to pre-industrial values (RCP4.5)^12^. Please refer to Kravitz *et al*.^11^ for the schematic of radiative forcings in G4. This rate of SO_2_ injection is equivalent to one Mount Pinatubo eruption every 4 years, and was designed to reduce the global-mean temperature to about 1980 values^11^. The background scenario in G4 is RCP4.5, in which greenhouse gas emissions peak at around 2040 and then decline, as this was thought to be a plausible concentration pathway with greenhouse gas emission mitigation implemented. Nonetheless, just as the RCP scenarios^13^, the G4 scenario is chosen to be illustrative only. We do not suggest a real-world application would be likely to follow this pathway.

Conventional detection and attribution estimates the amplitudes, or scaling factors, of model-simulated responses to different forcings or different groups of external forcings in the observations, with the null hypothesis of unforced, internal climate variability. This is done by regressing the observations against the model-simulated responses, or fingerprints, taking into account unforced variability in the observations and sampling uncertainty in the simulated responses. Assuming fingerprints of different external forcings, **x**_*i*_, are linearly additive and independent of internal variability, *u*_0_, the total least squares (TLS) multi-variate detection model^14^ is as follows:


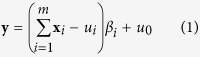


where **y** is the observations, *u*_*i*_ is the sampling noise in **x**_*i*_, *m* is the total number of fingerprints and *β*_*i*_ is the scaling factor of the *i*^th^ fingerprint to be estimated. If the solution *β*_*i*_ and its two-tailed confidence interval, usually its 5 to 95 percentiles, include zero, then the null hypothesis that the *i*^th^ fingerprint is absent from the observations cannot be ruled out at P_<0.1_. Rejection of this null hypothesis, i.e. *β*_*i*_ and its uncertainty range differ from zero, implies detection of the *i*^th^ fingerprint at the 10% level.

The Intergovernmental Panel on Climate Change (IPCC) have used this multi-variate detection method to robustly demonstrate human influence on the climate system over the last few decades^15^,^16^. We explore how this method could be used in a new way for detecting geoengineering signals. Using pseudo-observations (observations mimicked by climate model simulations) and cross-comparing them with simulations from other models, the time horizon over which SAI detection would become possible in annual-mean global-mean SAT time series from the start of deployment (taken in G4 to be in 2020) is estimated to the nearest 5 years. This metric is referred to as ‘SAI detection horizon’ hereafter.

As an alternative to the conventional, multi-variate method, Bürger and Cubasch^17^ have used a non-stationary detection method to study the detectability of sulphate aerosol geoengineering. The major difference between these two methods lies in their null hypotheses. Applied to the case of geoengineering in G4, the multi-variate method attempts to detect the SAI and RCP4.5 signals simultaneously against the climate system’s internal variability, whereas the non-stationary method attempts to detect the SAI signal against a gradual warming background caused by RCP4.5 forcings and internal variability. Using the non-stationary method and a trend-based data filter, Bürger and Cubasch found that spatial and spatio-temporal SAI signals would become detectable in temperature and precipitation after just a few years of sulphur dioxide injection in G4. Here we also apply this new method to detect the influence of SAI on future SAT pseudo-observations. We compare the SAI detection horizons estimated using these two methods, in conjunction with three different filtering procedures for noise removal in the data.

## Results

### Summary of detection methods and filters used

Table 1 summarises the 5 variations of TLS detection that are applied to detect the SAI signal in future annual-mean global-mean SAT time series in our study. Each of them is a unique combination of a detection method and a data filtering technique. Averaging and filtering raw observations and climate model simulations is often used in detection and attribution studies to increase the signal-to-noise ratio and, thus, the chance of successful detection^18^,^19^. In addition to annual mean and global averaging, a mean-based *C*0 and a trend-based *C*1 filter are used in 4 of our experiments, so that the effect of data filtering alone on the detection results can be investigated.

*C*0 is a moving filter that estimates the climate at a certain year from the mean climate of its previous *N* years. *N* is equal to 14 throughout our study. The estimation is based on the past because future information that is not available would be needed otherwise. Application of the 14-year-wide *C*0 filter results in a smoother time series that has a lag of approximately 7 years from the true mean climate^17^. On the other hand, the *C*1 filter estimates the climate in any given year from the trend of its previous *N* years (*N* = 14 in this study). The trend is estimated by using ordinary least squares regression. A 14-year-wide *C*1 filter does not result in an obvious phase shift from the true mean climate, but nonetheless produces a noisier time series than *C*0^17^. In addition to data smoothing, these two filters extract climate signals and deal with the short-term non-stationarity arising from abrupt geoengineering deployment, both of which are necessary procedures in the non-stationary detection approach.

*N* = 14 is found to be the optimal width for both filters, for capturing the short-term climate response to the SAI shock in G4 and retaining the temporal shape of the data without a large time delay^17^. A longer filter period, e.g. 30 years, results in a much smoother time series but also a time lag of around 15 years even with the C1 filter. We completed our own sensitivity test to confirm this result and agree with the conclusions of Bürger and Cubasch.

As opposed to the non-stationary method, the application of the *C*0 or the *C*1 filter is not necessary for the multi-variate method to work. We nevertheless also use them in conjunction with multi-variate detection to reduce noise in the observations and model simulations while retaining their temporal shapes, and to facilitate fairer comparison between the performance of the two detection methods.

### Detection using the multi-variate method and no filter (TfC0)

The time horizons over which SAI-forced changes would become detectable in annual-mean global-mean SAT in the G4 scenario estimated with TfNo are shown in Table 2. Each row represents pseudo-observations from a plausible geoengineering realisation, and each column corresponds to a different choice of climate model for generating fingerprints for detection purposes. The climate models included in this study are HadGEM2-ES^20^, CSIRO-Mk3L-1-2^21^, CanESM2^22^, BNU-ESM^23^ and MIROC-ESM^24^ (see Supplementary Table S1 for their modelling groups). The number in brackets in each column indicates the number of members over which ensemble mean is taken when estimating fingerprints from the simulations. BNU-ESM and MIROC-ESM have 1 ensemble member available each, while the rest of the models have 3 ensemble members in their G4 simulations. Using only 1 member to generate fingerprints is likely to result in insufficient separation of the forced signal from climate variability, and is not recommended^14^,^25^. However, its sensitivity to different detection methods is still useful to study in this paper.

There are 44 pseudo-observation model comparisons in total. Comparisons between pseudo-observations and fingerprints that are generated from the same climate model are excluded from the results. This is to avoid biases as the scaling factors estimated in these comparisons are very close to 1, as one would expect. These scaling factors nonetheless prove the credibility of the detection algorithm used.

For each comparison, the length of the input SAT time series in the detection algorithm is varied from 5 to 30 years at 5-year intervals. All time series start at year 2020, the year at which SAI is implemented in the geoengineering scenario and G4 simulations begin. The first time that the estimated scaling factor and its uncertainty range for the SAI fingerprint exclude zero is taken as the detection horizon.

At least 25 years of observations are needed for successful SAI detection in more than half of the studied comparisons. In particular, more than 30 years are needed in the combinations where the first ensemble member of MIROC-ESM is used as pseudo-observations, regardless of the model used for simulating fingerprints. This is because MIROC-ESM has the weakest global-mean cooling response to SAI among the models. The results suggest that SAI detection in this scenario will be challenging using the multi-variate detection approach and no filter, especially if future observations follow the MIROC-ESM trajectory.

**Figure 1:**
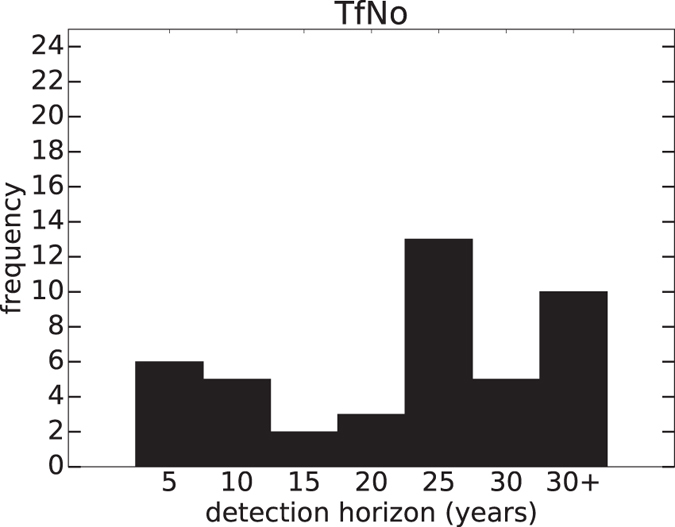
Distribution of the detection horizons estimated with TfNo. For most of the combinations, more than 25 years are required for SAI detection.

Figure 1 shows the distribution of the results from Table 2. Despite being spread out across different horizons, it can be seen that most comparisons require more than 25 years for SAI detection. This indicates that although swift cooling is generally expected from SAI, and is indeed observed in all of the model-simulated G4 time series, a longer time period would be required to robustly detect its influence if TfNo is to be used.

### Sensitivity of detection horizon to different filters

**Figure 2:**
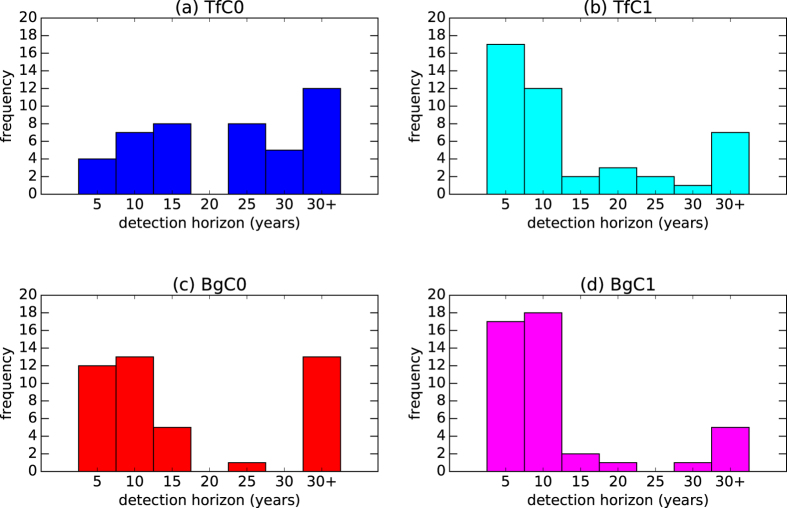
Distributions of the estimated time horizon over which the SAI influence would become detectable in the temporal pattern of global-mean SATs after 2020 by using the (a) TfC0, (b) TfC1, (c) BgC0 and (d) BgC1, respectively, at the 10% level. The results are more sensitive to the choice of filter than to the detection methods alone.

Figure 2(a) and (b) show the distributions of detection horizons estimated for exactly the same 44 pseudo-observation model comparisons as above using TfC0 and TfC1, respectively. Compared to not filtering the observations, model-simulated fingerprints and the control at all, detection of the SAI influence on global-mean SAT becomes possible within the first 15 years of SAI deployment in more comparisons when the *C*0 filter is used. However, the detection horizons are still spread out with a peak at more than 30 years. This peak is mostly contributed from comparisons that involve CSIRO-Mk3L-1-2, in which solar irradiance reduction is used instead of aerosol prescription or injection.

The residual consistency test^14^,^26^ fails more often with the *C*0 filter than when no filter is applied, even though exactly the same comparisons are being studied throughout. This means that when the *C*0 filter is used, the weighted sum of squared residuals of regression is inconsistent with the model-simulated noise variance in more comparisons than when no filtering is done. This has affected some of the estimated detection horizons, as detection cannot be claimed when the control simulation of climate variability, and hence uncertainty estimates on the scaling factors, is distrusted. Inconsistencies in model responses on longer timescales may have contributed to the increased number of failures in the residual check.

The application of the *C*1 filter alone, however, results in a significant shift in the estimated detection horizons to shorter timescales, as can be seen in the comparison between Figs 1 and 2(b). The SAI influence would become detectable at the 10% level within the first decade of SAI deployment in 29 of the 44 studied combinations. This suggests that even with the same null hypothesis, pseudo-observations and set of climate models, the detection results are highly sensitive to the change from not filtering the data to using a trend-based filter to define the input time series. The small peak at more than 30 years on Fig. 2(b), again, mostly comes from comparisons that involve CSIRO-Mk3L-1-2.

### Sensitivity of detection horizon to different methods

The distributions of detection horizons estimated by using BgC0 and BgC1 are shown in Fig. 2(c) and (d), respectively. Switching from the conventional, multi-variate method to the non-stationary method results in a slight shift in detection horizons from longer timescales to within a decade, as can be seen by comparing Fig. 2(a) with (c), and Fig. 2(b) with (d). However, the results are not as sensitive to detection methods as they are to the choice of filter. The residuals mismatch problem seen with TfC0 persists when BgC0 is used. With the *C*1 filter applied, the distribution of detection horizons estimated with the non-stationary method is very similar to that estimated by using the multi-variate method, both having peaks at the first 5 and 10 years of SAI deployment.

The early detectability, i.e. within a decade, of SAI found in the BgC1 experiment is consistent with the conclusion drawn by Bürger and Cubasch^17^, even though spatial information is not included in our study. Furthermore, the similarity in the distributions of detection horizon estimated by using TfC1 and BgC1 confirms the robustness of such a finding, regardless of whether the multi-variate or the non-stationary detection method is used.

## Discussion

By applying two variants of total least squares detection and three different data filtering procedures to annual-mean global-mean near-surface air temperature time series in the G4 scenario, we detect sulphate aerosol geoengineering signals after 2020 at the 10% level. We have shown the high sensitivity of sulphate aerosol detectability to the choice of filter for data smoothing. Filtering pseudo-observations, model-simulated fingerprints and the background climate with a trend-based filter (*C*1) results in earlier detection, compared to when a mean-based filter (*C*0) or no filter is used. This is because the *C*1 filter effectively removes noise while keeping the temporal shape of the raw data without an obvious lag, like temporal mean does.

The use of the conventional, multi-variate detection method in conjunction with a 14-year wide *C*1 filter (TfC1) results in successful detection of the sulphate aerosol signal within a decade of geoengineering deployment, in around 66% of the studied pseudo-observation model pairs. This result is comparable to that of Bürger and Cubasch^17^, even though a significantly different null hypothesis and additional spatial information are used in their study. Our results, therefore, confirm our ability to detect the influence of injected sulphate aerosols on near-surface air temperature within 10 years of geoengineering deployment regardless of the null hypothesis chosen, should the future observations follow the G4 projections, and the same climate variable, models and filtering technique are used.

In comparison to the filtering technique, the detection method alone has a smaller effect on geoengineering detectability. Nonetheless, the combined use of the non-stationary method and the 14-year wide *C*1 filter (BgC1) results in early detectability in the highest number of studied comparisons. The sulphate aerosol signal becomes detectable within 10 years of deployment in around 80% of the studied comparisons. This is consistent with Bürger and Cubasch’s results. It is, therefore, evident that the early geoengineering detectability found in their study is mainly the result of a good choice of filter, rather than the null hypothesis that includes a forced warming trend.

Whilst neither the multi-variate method nor the non-stationary method is incorrect, there are certain advantages of using TfC1 over BgC1. Firstly, the multi-variate detection method has been very widely used for detecting and attributing climate change in many different climate variables. On the other hand, there is no proven credibility of the non-stationary method except for its application to surface temperature and precipitation in hypothetical geoengineered worlds. Secondly, the multi-variate method allows simultaneous detection of climate responses to different forcings or groups of forcings. This means the detection of anthropogenic warming before geoengineering could serve as a check, as climate warming should have been robustly detected before geoengineering is considered and deployed. Indeed, with the use of TfC1, the RCP4.5 signal is detected before or at the same time as the geoengineering signal in almost all of our studied comparisons. This is a useful check that the non-stationary method cannot provide.

Moreover, although not included in this study, one could attribute a change in climate observable with geoengineering to individual factors such as solar activity, volcanic eruptions, or many other climate forcings simultaneously only with the use of the multi-variate method. Understanding how much of the observed changes is due to the intentionally injected sulphate aerosols relative to other external forcings would be crucial in the event of deployment, especially if we wanted to manage geoengineering via explicit feedback^9^,^10^. An attribution approach could also help us to understand by what extent the climate models that we use to calculate the amount of SAI needed for achieving certain climate goals are underestimating or overestimating the real world’s responses to the injected sulphate aerosols. Future work could investigate this using the multi-variate detection method.

Only data from 2020 onward have been included in this study, as SAI only starts in 2020 in G4 and this is the point at which the G4 simulations diverge from RCP4.5. In reality, any detection in the real-world may also make use of observations and model simulations prior to deployment. To investigate the impact of prior observations we also performed sensitivity studies in which we extended the time series backwards. This means adding historical and RCP4.5 data to the pseudo-observation and fingerprint time series. It potentially leads to noise contamination in the non-stationary method^17^ and, therefore, has been avoided in the main study for detection method comparison. In the sensitivity experiment where a diagnostic beginning in 2000 and TfC1 were used, 10 comparisons would need 5 years of geoengineering observations for SAI detection at the 10% level, while 14 other comparisons would require 10 years to do so. The drop in the number of successful detections in the first 5 years of SAI implementation may be explained by signal degeneracy^15^,^19^: the extended 2000–2024 G4 and RCP4.5 fingerprints are so similar that amplifying one of them while diminishing the other may explain the pseudo-observations just as well as the other way round.

Nevertheless, allowing the use of pre-deployment data is another advantage of the multi-variate method over the non-stationary method and may result in earlier detection when less pre-deployment data or further spatial information is included (although this is beyond the scope of the present study). Future work could then investigate how much pre-deployment data would be optimal for SAI detection using the multi-variate method.

Given the high sensitivity of the geoengineering detection horizon to the choice of filter, further work could also test the effect of the width of a moving filter on the results. Also, our experiments are highly idealised to assume observations at evenly distributed grid points. A more realistic study could use data at points that are available in the current observational system only. Future work could also attempt to detect the geoengineering signal at other scales or in other climate variables such as precipitation and reflected shortwave radiation. These variables are expected to respond to the radiative forcing changes associated with geoengineering aerosols, but detection may be more challenging due to a larger spread in model responses and lower signal-to-noise ratio, especially at regional scales.

Finally, this study has heavily relied on hypothetical climate scenarios and climate model simulations, as SAI has not happened in reality at large scales. A background climate following RCP4.5 has been assumed in our study, but stabilising radiative forcing to 4.5 Wm^−2^ above pre-industrial levels by 2100 would require greenhouse gas emission mitigation^16^,^27^. Radiative forcings in the real world are uncertain, unlike the known forcings used in the RCP4.5 simulations. The lack of perfect knowledge of radiaitve forcings particularly at short time periods would add uncertainty to the detectability of SAI in a real-world application. Sulphate aerosols have been injected or prescribed in the climate models at a fixed rate of 5 Tg yr^−1^, but this scenario may not be optimal or achievable, and is only one of the many possible ways of geoengineering implementation^28^,^29^. Therefore, the robust early detectability of sulphate aerosol injection on global-mean temperature found in this study does not imply geoengineering of this kind should be deployed. Whether or not it will be needed depends highly on future greenhouse gas concentrations and climate trajectories, countries’ efforts to mitigate greenhouse gas emissions and their climate objectives. Also, the unintentional effects of SAI on the climate system, its socio-economic impacts, and associated ethical and political complications, all of which are out of the scope of this study, should be taken into serious consideration in future climate policymaking^30^.

## Methods

### Data sets

The GeoMIP^11^ was designed to establish a coordinating framework for modelling groups to explore possible climate responses to various SRM methods. There were 9 models participating in the G4 experiment^31^. 7 of them were fully coupled atmosphere-ocean general circulation models (AOGCMs), and 2 were coupled chemistry-climate models. Using output from the 7 AOGCMs, a recent study^32^ estimated the difference between 2030–2069 and 2010–2029 global-mean SAT under the RCP4.5 scenario to be 0.81 ± 0.21 °C. The difference would reduce to 0.28 ± 0.31 °C in the comparison between 2030–2069 G4 and 2010–2029 RCP4.5 global-mean SATs.

**Figure 3:**
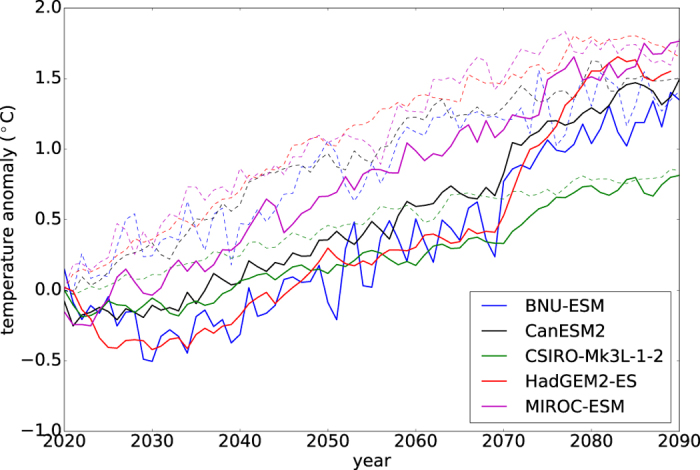
Ensemble-mean annual-mean global-mean SAT anomaly (relative to the corresponding 2020 levels) time series in G4 (thick solid line) and RCP4.5 (dashed line) from BNU-ESM, CanESM2, CSIRO-Mk3L-1-2, HadGEM2-ES and MIROC-ESM, respectively.

SAT output from 5 of the participating AOGCMs, namely HadGEM2-ES, CSIRO-Mk3L-1-2, BNU-ESM, MIROC-ESM and CanESM2, were used in this study. Figure 3 shows the time series of ensemble-mean annual-mean global-mean SAT anomalies relative to the corresponding 2020 levels in G4 (thick solid lines) and RCP4.5 (dashed lines) from these models. Temperatures in G4 are lower than that in RCP4.5 throughout the deployment period in all of the models. BNU-ESM and MIROC-ESM have nosier time series as they have only 1 ensemble member each, while the rest of the models have 3. GISS-E2-R^33^ and MIROC-ESM-CHEM^24^ were not included because of incorrect initialisation in G4^17^ and unavailable pre-industrial output for download at the time of study, respectively. The 2 coupled chemistry-climate models have prescribed sea surface temperature, making them non-comparable to other models.

BNU-ESM has an imperfect initialisation in G4 which results in a slight discrepancy between RCP4.5 and G4 pre-2020 temperatures. However, given their very similar pre-2020 climatologies, BNU-ESM was retained in the study (Duoying Ji, personal communication, 2015). Although CSIRO-Mk3L-1-2 uses solar irradiance reduction instead of prescribed or injected stratospheric sulphate aerosols, it was retained in this global-mean study to maintain a sizeable sampling pool.

### Data processing

The global-mean annual-mean SAT time series used throughout our study were produced by taking the area-weighted global average from temperatures at all available grid points, followed by calculating the annual means.

For TfNo, TfC0 and TfC1, the pseudo-observations, **y**_Tf_, was a time series of annual-mean global-mean G4 SAT anomalies with respect to the ensemble member’s own 2006–2019 SAT mean that was filtered according to the experiment. The time series started in 2020 and the filter window width was 14 years. The model-simulated fingerprints, **x**_G4_ and **x**_RCP_, were time series of ensemble-mean annual-mean global-mean G4 and RCP4.5 SAT anomalies with respect to the model’s corresponding 2006–2019 SAT mean, respectively. They were filtered in exactly the same way as was **y**_Tf_. All of these time series had the same length, which depended on the future time period of interest.

Unforced pre-industrial simulations (piControl) from HadGEM2-ES, CSIRO-Mk3L-1-2, GISS-E2-R, CanESM2, BNU-ESM and MIROC-ESM were used to estimate internal climate variability. The power spectra of all model’s temperature variability were found to be comparable to those of HadCRUT4^34^ and GISTEMP^35^ on the timescales of 5 to 30 years individually (see Supplementary Fig. S1, cf. Gillett *et al*.^36^). A linear trend was removed from the annual-mean global-mean piControl time series if it had a linear shift (BNU-ESM and MIROC-ESM), while a mean was removed if there was no obvious trend in the piControl time series (HadGEM2-ES, CSIRO-Mk3L-1-2, GISS-E2-R and CanESM2). Each of the model’s standardised piControls was then split into segments of the length of **y**_Tf_. The same diagnostic as was applied to **y**_Tf_ was applied to every segment here. Half of these segments were used for optimisation, while the other half were used for hypothesis testing in the detection and attribution algorithm. The number of degrees of freedom of internal variability estimation was the number of segments used for hypothesis testing. The same multi-model internal variability dataset was used irrespective of which model was used to represent pseudo-observations and which was used to generate fingerprints.

For BgC0 and BgC1, **y**_Bg_ was a time series of annual-mean global-mean G4 SAT time series starting from 2020, anomalised and filtered in the same way as described above, but with a mean RCP4.5 background climate removed^17^. This was achieved by subtracting the corresponding mean SAT over 114 RCP4.5 simulations from each value in the G4 time series. A list of the climate models used to estimate the RCP4.5 background climate can be found in Supplementary Table S1. The ensemble-mean model-simulated response of interest in this case, **x**_SAI_, was processed in the same way as **y**_Bg_.

The 114 RCP4.5 simulations were processed in exactly the same way as was **y**_Bg_ to estimate the non-stationary background climate. Similar to the treatment to piControl, these simulations were divided into two groups for optimisation and hypothesis testing. This background climate estimation was fixed across the pseudo-observation model comparisons.

### Optimal detection

Equation 1 was solved using the TLS function in the Environment Canada’s Optimal Fingerprint (ECOF) package (Yang Feng, personal communication, 2015). In the optimal detection algorithm, the pseudo-observations and fingerprints described above were projected onto empirical orthogonal functions (EOFs) of the control (eigenvectors of the climate noise covariance matrix) and weighted by their inverse singular values so that the signal-to-noise ratio was maximised^26^. Since the number of independent piControl segments for optimisation was lower than the rank of the pseudo-observations, the inverse noise covariance matrix necessary to solve Equation 1 was estimated from a truncation of projections onto the leading EOFs^14^,^26^.

Both optimisation and hypothesis testing in the detection algorithm require knowledge of the characteristics of internal climate variability. This was obtained from the piControl simulations, as mentioned in the previous section. While modelled internal variability is not expected to completely reproduce variability in the real world, it should be a realistic representation at the truncated scales retained in the analysis. The maximum reliable truncation was chosen via a residual consistency test^14^,^26^, in which the weighted sum of squared residuals of regression was compared to the model-simulated noise variance via an *F* test. For each pseudo-observation model comparison, the highest truncation at which the *F* test probability fell within the 5–95% range while the corresponding estimated scaling factor varied little with truncation was selected. The detection result then depended on whether the uncertainty range on the scaling factor at the selected truncation included zero or not.

### Linear transformation of scaling factors

For the two-fingerprint experiments, TLS regression against the G4 and RCP4.5 simulations, **x**_G4_ and **x**_RCP_, was used instead of **x**_SAI_ and **x**_RCP_, due to a lack of SAI-only simulations. We extracted the scaling factors for the SAI and RCP4.5 forced signals from that of **x**_G4_ by transforming scaling factors after regression^18^. Assuming **x**_G4_ = **x**_SAI_ + **x**_RCP_:


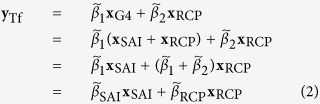


where 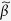
’s are best-estimate scaling factors. Linear transformation in matrix form is thus:


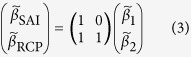


Equation 3 was used to estimate both the best estimates and uncertainty ranges of scaling factors for hypothesis testing in TfNo, TfC0 and TfC1.

## Additional Information

**How to cite this article**: Lo, Y. T. E. *et al*. Detecting sulphate aerosol geoengineering with different methods. *Sci. Rep.* **6**, 39169; doi: 10.1038/srep39169 (2016).

**Publisher's note:** Springer Nature remains neutral with regard to jurisdictional claims in published maps and institutional affiliations.

## Supplementary Material

Supplementary Information

## Figures and Tables

**Table 1:** Detection methods and filters that are used to test the sensitivity of sulphate aerosol geoengineering detection.

Name	Fingerprints	Filter	Background climate
TfNo	SAI and RCP4.5	None	Multi-model pre-industrial simulations
TfC0	SAI and RCP4.5	*C*0	Multi-model pre-industrial simulations
TfC1	SAI and RCP4.5	*C*1	Multi-model pre-industrial simulations
BgC0	SAI	*C*0	Multi-model RCP4.5 simulations
BgC1	SAI	*C*1	Multi-model RCP4.5 simulations

TfNo, TfC0 and TfC1 are the conventional, two-fingerprint method with no filter, *C*0 filter and *C*1 filter applied, respectively. BgC0 and BgC1 are Bürger and Cubasch’s non-stationary method with the *C*0 filter and *C*1 filter applied, respectively. Filter width is 14 years where applicable.

**Table 2:** Estimated minimum number of years that are needed for the SAI influence to become detectable in annual-mean global-mean SAT from deployment in 2020, using the TfNo method at the 10% level.

	HadGEM2-ES (3)	CSIRO-Mk3L-1-2 (3)	CanESM2 (3)	BNU-ESM (1)	MIROC-ESM (1)
HadGEM2-ES r1	—	25	10	15	25
HadGEM2-ES r2	—	5	10	5	25
HadGEM2-ES r3	—	25	30	5	30+
CSIRO-Mk3L-1-2 r1	25	—	15	30+	25
CSIRO-Mk3L-1-2 r2	25	—	25	30+	25
CSIRO-Mk3L-1-2 r3	30+	—	30	30	30
CanESM2 r1	5	30+	—	30	20
CanESM2 r2	25	30+	—	20	25
CanESM2 r3	20	10	—	10	25
BNU-ESM r1	5	5	10	—	25
MIROC-ESM r1	30+	30+	30+	30+	—

There are 44 pseudo-observation (in rows) model (in columns) comparisons in total. r1, r2 and r3 stand for the first, second and third G4 realisation, respectively. Numbers in brackets indicate the number of members over which ensemble mean is taken in the model-simulations.
