# Corrigendum: Detecting sulphate aerosol geoengineering with different methods

**DOI:** 10.1038/srep46905

**Published:** 2017-09-15

**Authors:** Y. T. Eunice Lo, Andrew J. Charlton-Perez, Fraser C. Lott, Eleanor J. Highwood

Scientific Reports 6: Article number: 39169; 10.1038/srep39169 published online: 12 15 2016; updated: 09 15 2017.

This Article contains errors. In Equation 1,
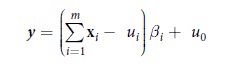


should read:
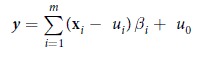


In the Results section, the subheading ‘Detection using the multi-variate method and no filter (TfC0)’ should read:

‘Detection using the multi-variate method and no filter (TfNo)’.

