# Supplementary material for: Detecting sulphate aerosol geoengineering with different methods
Source: Sci Rep. 2016 Dec 15;6:39169. doi: 10.1038/srep39169 (PMC5156937; doi:10.1038/srep39169)

Detecting sulphate aerosol geoengineering with different methods

Y. T. Eunice Lo[1,*], Andrew J. Charlton-Perez[1], Fraser C. Lott[2] and Eleanor J. Highwood[1]

[1] Department of Meteorology, University of Reading, Reading RG6 6BB, UK
[2] Met Office Hadley Centre, FitzRoy Road, Exeter EX1 3PB, UK
[*] y.t.e.lo@pgr.reading.ac.uk

Supplementary Table S1. GeoMIP and CMIP5 model output used in the study and the modelling groups that provided them.

Model name	Modelling group	Output used
ACCESS1.0<br>ACCESS1.3	Commonwealth Scientific and Industrial Research Organization (CSIRO) and Bureau of Meteorology (BOM), Australia	RCP4.5
BCC-CSM1.1<br>BCC-CSM1.1(m)	Beijing Climate Center, China Meteorological Administration	RCP4.5
BNU-ESM	College of Global Change and Earth System Science, Beijing Normal University	G4, RCP4.5, piControl
CanESM2	Canadian Centre for Climate Modelling and Analysis	G4, RCP4.5, piControl
CCSM4	National Center for Atmospheric Research	RCP4.5
CESM1(BGC)<br>CESM1(CAM5)<br>CESM1(WACCM)	Community Earth System Model Contributors	RCP4.5
CMCC-CM<br>CMCC-CMS	Centro Euro-Mediterraneo per I Cambiamenti Climatici	RCP4.5
CNRM-CM5	Centre National de Recherches Météorologiques / Centre Européen de Recherche et Formation Avancée en Calcul Scientifique	RCP4.5
CSIRO-Mk3.6.0	Commonwealth Scientific and Industrial Research Organization in collaboration with Queensland Climate Change Centre of Excellence	G4, RCP4.5, piControl
EC-EARTH	EC-EARTH consortium	RCP4.5
FIO-ESM	The First Institute of Oceanography, SOA, China	RCP4.5
GFDL-CM3<br>GFDL-ESM2G<br>GFDL-ESM2M	NOAA Geophysical Fluid Dynamics Laboratory	RCP4.5
GISS-E2-H	NASA Goddard Institute for Space Studies	RCP4.5

GISS-E2-R		RCP4.5, piControl
HadGEM2-AO	National Institute of Meteorological Research/Korea Meteorological Administration	RCP4.5
HadGEM2-CC	Met Office Hadley Centre (additional HadGEM2-ES realizations contributed by Instituto Nacional de Pesquisas Espaciais)	RCP4.5
HadGEM2-ES		G4, RCP4.5, piControl
INM-CM4	Institute for Numerical Mathematics	RCP4.5
IPSL-CM5A-LR<br>IPSL-CM5A-MR<br>IPSL-CM5B-LR	Institut Pierre-Simon Laplace	RCP4.5
MIROC-ESM	Japan Agency for Marine-Earth Science and Technology, Atmosphere and Ocean Research Institute (The University of Tokyo), and National Institute for Environmental Studies	G4, RCP4.5, piControl
MIROC-ESM-CHEM		RCP4.5
MIROC5	Atmosphere and Ocean Research Institute (The University of Tokyo), National Institute for Environmental Studies, and Japan Agency for Marine-Earth Science and Technology	RCP4.5
MPI-ESM-MR<br>MPI-ESM-LR	Max-Planck-Institut für Meteorologie (Max Planck Institute for Meteorology)	RCP4.5
MRI-CGCM3	Meteorological Research Institute	RCP4.5
NorESM1-M<br>NorESM1-ME	Norwegian Climate Centre	RCP4.5

Supplementary Figure S1. Power spectral densities of linearly detrended 1981-2015 annual-mean global-mean temperatures from HadCRUT4 (green line) and GISTEMP (blue line), and the mean spectral density found from 35-year segments of the model's pre-industrial control simulation (dashed line). The grey region shows the range of spectral densities found from the control segments.

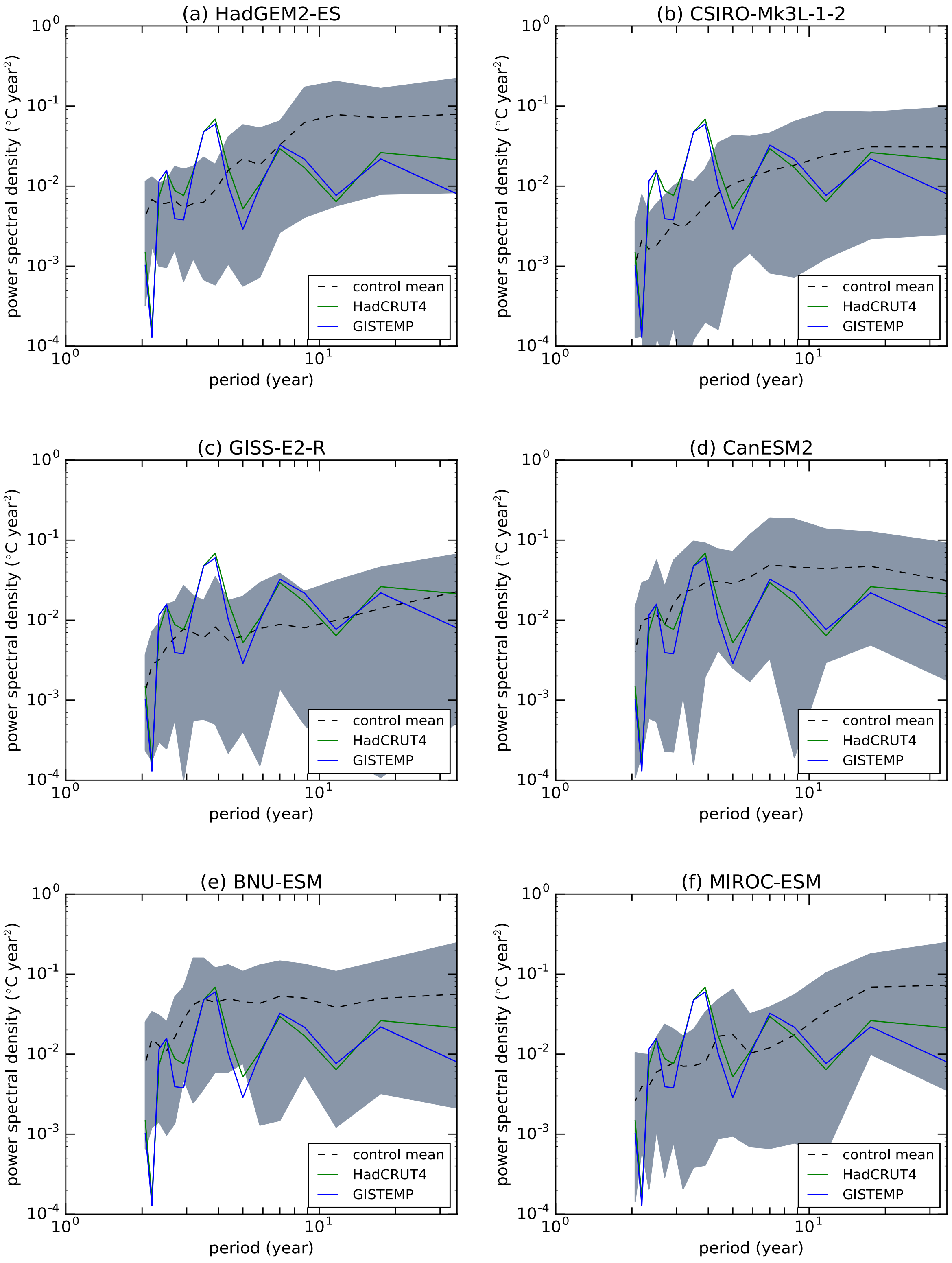

Supplement: Supplementary Information [file srep39169-s1.pdf]
